# Heredity of Tuberculosis

**Published:** 1889-10

**Authors:** 


HEREDITY OF TUBERCULOSIS.—The importance now attaching to every phase of the study of Tuberculosis renders the following, taken from the *Sanitarian*, of interest:

Hutinel, of the *Hôpital des Enfants Malades*, has published a communication on the Heredity of Tuberculosis, of which the conclusions are as follows:

As for the direct transmission of the germ from the parents, we are warranted in affirming that communication by the father is not proved, and is very problematical; transmission by the mother may take place, but it is extremely rare.

Heredity, nevertheless, exists; for if the offspring of phthisical parents are not born tuberculous, they are *tuberculizable;* they may not, it is true, have brought with them the germ from their birth, but they have inherited a culture soil (*terrain*) favorable to its development.

In accordance with these conclusions, which are in harmony with a multitude of clinical observations, Langerhaus, who has long practised in Madeira, has noted that among the individuals not phthisical that have come to that island for a certain number of years, only those born of tuberculosis parents become infected by the numerous phthisical patients resorting to that island every year. Of every ten individuals with phthisical parentage, one died of tuberculosis, while the proportion of those becoming consumptive with no hereditary predisposition has been as one to sixty-eight.

